# Differences in the performance of NK1R−/− (‘knockout’) and wildtype mice in the 5‑Choice Continuous Performance Test

**DOI:** 10.1016/j.bbr.2015.10.045

**Published:** 2016-02-01

**Authors:** Ashley J. Porter, Katharine Pillidge, S. Clare Stanford, Jared W. Young

**Affiliations:** a Department of Neuroscience, Physiology and Pharmacology, University College London, Gower Street, London WC1E 6BT, UK; b Department of Psychiatry, University of California San Diego, La Jolla, CA, USA; c Research Service, VA San Diego Healthcare System, San Diego, CA, USA

**Keywords:** ADHD, False alarms, Inattentiveness, NK1 receptor, Perseveration, Premature responses, 5-Choice Continuous-Performance Test, Impulsivity, Motor disinhibition

## Abstract

Mice lacking functional NK1 (substance P-preferring) receptors typically display excessive inattentiveness (*omission errors*) and impulsivity (*premature responses*) when compared with wildtypes in the 5-Choice Serial Reaction-Time Test (5-CSRTT). These abnormal behaviours are analogous to those seen in humans suffering from Attention Deficit Hyperactivity Disorder (ADHD). Here we used the 5-Choice Continuous‑Performance Test (5C-CPT) to ascertain whether NK1R−/− mice also display excessive *false alarms* (an inappropriate response to a ‘*no-go’* signal), which is another form of impulsive behaviour. NK1R−/− mice completed more trials than wildtypes, confirming their ability to learn and carry out the task. At the start of Stage 1 of training, but not subsequently, they also scored more *premature responses* than wildtypes. When the mice were tested for the first time, neither *false alarms* nor *premature responses* was higher in NK1R−/− mice than wildtypes but, as in the 5-CSRTT, the latter behaviour was strongly dependent on time of day. NK1R−/− mice expressed excessive *perseveration* during all stages of the 5C-CPT. This behaviour is thought to reflect compulsive checking, which is common in ADHD patients. These findings point to differences in the 5-CSRTT and 5C-CPT protocols that could be important for distinguishing why the cognitive performance and response control of NK1R−/− mice differs from their wildtypes. The results further lead to the prediction that ADHD patients with polymorphism of the *TACR1* gene (the human equivalent of *Nk1r*) would express more *perseveration*, but not *false alarms*, in Continuous Performance Tests when compared with other groups of subjects.

## Highlights

• We compared the behaviour of NK1R−/− mice and wildtypes in the 5-Choice Continuous Performance Test.
• NK1R−/− mice did not express excess impulsivity (premature response or false alarms) in this test.
• NK1R−/− mice expressed excessive perseveration, which is common in ADHD.
• The findings point to a behavioural phenotype for ADHD patients with polymorphism of the *TACR1* gene.

## 1 Introduction

Attention Deficit Hyperactivity Disorder (ADHD) is a highly prevalent illness, which persists into adulthood in the majority of cases [1], [2]. In childhood, patients suffer from hyperactivity, inattention and impulsivity, which contribute to difficulties at school and everyday life [3], [4]. As adults, ADHD patients commonly experience co-morbid social, occupational and health problems [3], [5].

A limited number of treatments for ADHD are available, but these were not targeted at the neural mechanisms underlying the disorder because these mechanisms have yet to be identified. Amongst the candidates thought to cause, or increase vulnerability to, ADHD is polymorphism(s) of the (human) *TACR1* gene [6], [7], which is equivalent to the *Nk1* (substance P-preferring) receptor gene in rodents. Delineating a role for the *Nk1r* gene in behaviours relevant to ADHD could enable more targeted development of drug treatments for this disorder.

Mice with functional ablation of the *Nk1r* gene have been generated (‘NK1R−/−’) [8] and we have reported previously that the males are hyperactive in a range of environmental contexts: *e.g.* an activity meter [9]; a light/dark exploration-box [6], [10], [11]; and in their home‑cage [12]. Also, when assessed for the first time in a widely-used test of sustained attention, the 5-Choice Serial Reaction-Time task (5‑CSRTT) [13], [14], NK1R−/− mice typically respond before stimuli appear (*premature response*, a form of motor impulsivity) and miss responding to stimuli (*omission error*, a measure of attention) [11], [12], [15], [16]. These behavioural abnormalities are arguably analogous to the hyperactivity, impulsivity and inattention seen in ADHD patients.

The inattention and response disinhibition of ADHD patients, however, is more commonly quantified using procedures such as the Conner's Continuous Performance Test (CPT) and the Test of Variables of Attention (TOVA: [17], [18], [19]). Both the CPT and TOVA include ‘target’ ('*go*​') trials, to which subjects should respond, and ‘non-target’ (‘*no*‑*go*’) trials, to which they should withhold any response (*false alarms,* in the breach) [19], [20], [21]. In the latter case, these tests differ from the 5‑CSRTT, which incorporates only (‘*go*’) targets that require responses. For this reason, a 5‑Choice Continuous‑Performance Test (5C-CPT), which includes both target and non-target trials, was developed to assess vigilance in mice [20], [21], [22], rats [23], [24] and humans [22], [25], [26]. A preclinical study relevant to ADHD has reported that both methylphenidate and atomoxetine, which are licensed to treat ADHD, improve attention and reduced response disinhibition in rats that are performing poorly in the 5C-CPT [27].

Another important feature of the 5C-CPT is that, by including non-target stimuli, it enables quantification of impulsivity in the form of both *premature responses* (as in the 5‑CSRTT) and *false alarm* responses (as in human CPTs). This differentiation is important because there is now extensive evidence that there are several aspects of impulsivity [28] and that different types of impulsivity recruit different neuronal networks [29], [30]: *i.e.* these measures are neuro-mechanistically dissociable [21]. A further difference between the two tests is that a progressive decrement in vigilance develops when rodents are tested in the 5C-CPT [20], [21], [23], which is rarely seen in the 5‑CSRTT.

Here, we further interrogated the putative association between the NK1 receptor and ADHD by comparing the performance of NK1R−/− mice and their wildtypes during training and testing in the 5C-CPT. We hypothesized that *Nk1r* ‘knockout’ mice would exhibit increased inattention and impulsivity (*premature responses* and *false alarms*), which are quantified in the 5C-CPT as lower *vigilance*.

## 2 Materials and methods

All procedures complied with the Animals (Scientific Procedures) Act (UK) [2010/63/EU] and had received local ethical approval at University College London.

### 2.1 Apparatus

The apparatus, described in detail elsewhere [15], was supplied by Med Associates (St. Albans, VT, USA) and was controlled by a Smart Ctrl Package 8IN/16OUT with an additional interface by MED-PC for Windows (Med Associates, St. Albans, VT, USA). The software was refined to incorporate a *no-go* signal (see: [20]).

### 2.2 Animals

All the mice were bred at University College London and housed in a facility at 21 ± 2 °C, 45 ± 5% humidity, with a 12/12 h light/dark cycle (lighting increased in steps from 07.00 to 08.00 h). We used twelve male wildtype mice (aged 6–7 weeks; weight: 30–34 g at the start of the study) and twelve male NK1R−/− mice of the same age-range (weight: 29–31 g). Inbred homozygous mice, rather than the (F2) offspring of heterozygous breeding pairs, were studied. This is because the incidence of *premature responses* in the 5-CSRTT (but not that of *omissions* or *hyperactivity* during the dark phase) depends on an interaction between a lack of *Nk1r* and breeding environment and is typically higher than their wildtypes only in inbred homozygotes [see: [12]]. The two genotypes shared the same background strain (129/Sv × C57BL/6J, crossed with outbred MF1 mice, many (more than 10) generations ago [8]). Wildtype mice were taken from two breeding pairs and NK1R−/− mice were taken from three breeding pairs and were group‑housed as littermates (2–5 per cage). The home‑cages incorporated environmental enrichment (cardboard tunnels and tissue for nesting material) and were cleaned twice weekly (bedding: 3Rs Bedding Pty., Ltd.). They were given free access to water throughout, but were fed a restricted diet (2018 Global Rodent Diet, Harlan) so as to maintain their body weight at 90% free-feeding weight. Every weekday, all the mice were weighed before training/testing in the 5C-CPT and fed between 16.00 and 17.00 h (after training/testing) with a quota of food determined by their body weight. At weekends, when there were no training or testing sessions, the mice were fed with 50% of their daily quota in the morning (between 09.00 and 11.00 h) and the remainder was given in the afternoon (between 16.00 and 18.00 h).

### 2.3 Training

At the start of the experiment, each mouse was assigned to one of four test chambers, counterbalancing for genotype, time of day (for training/testing) and home cage. This configuration was maintained throughout the experiment. Half the cohort was trained/tested in one of the morning sessions (three sessions were run between 10.00 and 12.00 h). The remainder were assigned to one of the afternoon sessions (three sessions were run between 13.00 and 15.00 h). Individual mice were trained/tested at the same time each day. All the behavioural data were captured and stored on-line. Each daily training session lasted for 120 trials or 30 min, whichever occurred first.

The animals carried out the training/test sessions with the house‑light switched off (unlike the 5‑CSRTT). After every correct trial, they were rewarded by delivery of an aliquot of sweetened milk (10 μL), which was available for 4 s. If the mice committed an *omission* error, commission error (*premature response* or *false alarm*), or incorrect response, the house‑light was turned on for 5 s, as a ‘punishment’, during which time a new trial could not be initiated (‘time out’).

During Stage 1 of training, the animals experienced only ‘*go*’ trials, which were delivered at a fixed interval (5 s; fixed ‘ITI’, as in the 5-CSRTT). In Stage 2, the *go* signal was delivered on a variable intertrial interval schedule (VITI: 3–7 s). Subsequent stages (3 and 4) incorporated *no-go,* as well as *go,* signals that were delivered with a VITI schedule: the ratio of *go:no*‑*go* signals was 2:1 during Stage 3 and 5:1 during Stage 4. The mice graduated from one stage of training to the next when they had satisfied the performance criteria for a minimum of three consecutive days.

After reaching the performance criteria in Stage 4 (‘baseline’: Table 1), each animal was tested on the following Friday in an extended series of trials (‘NI-1’). One difference between NI-1 and Stage 4 of training was that the number of trials was increased to 250 trials or 60 min, whichever was reached first. Another was that the ITIs were increased to 7–11 s. These were delivered in a random sequence, with the *go:no*‑*go* signals remaining at a 5:1 ratio. As a consequence, the signal parameters in NI-1 in this 5C-CPT combined an increase in the latency of the ITIs (reduced event‑rate) with their delivery in a random sequence (‘VITI’). This approach differs from the test session (NI-1) of the 5-CSRTT in two ways. First, in the 5-CSRTT, NI‑1 is the animals’ first experience of a VITI and, secondly, animals’ responses during the VITI and the prolonged ITI (LITI) tests are evaluated separately.

### 2.4 Statistical analysis of the data

The performance variables evaluated in this 5C-CPT include those already described for these mice when tested in the 5-CSRTT: namely, *%accuracy*, *%omissions*, *%premature responses*, *perseverative responses*, *latency to collect reward* and *latency to correct response* (see: [15]). However, the 5C-CPT provides additional measures including: the *probability of false alarms* (pFA: an index of response disinhibition); *hit rate* (HR: a measure of an animal’s overall response rate); *the latency to false alarms*; the *responsivity index* (RI: an index of an animal's tendency to carry out a motor response triggered by either a *go* or a *no*‑*go* light cue); and the *sensitivity index* (SI: an index of an animal’s sensitivity to *go versus no-go* stimuli). The calculation of these behaviours is described fully, elsewhere [20].

InVivoStat (version 2.3.0.0: [31]) was used to analyse the data. First, diagnostic plots were constructed to check for normality of the data‑set and equality of the sample variance. When necessary, the data were transformed (√ score or Log_10_(score + 1)) to optimise the homogeneity of variance across the experimental groups before proceeding with subsequent analyses. Mead’s resource equation was used to confirm that sample sizes were adequate to detect statistical significance.

Data for each of the Stages 1–4 of training were analysed separately. Those for Stage 1 (fixed ITI phase), were analysed using repeated measures ANOVA with ‘Stimulus Duration’ as the within-subjects factor and ‘Genotype’ and ‘Time-of-Day (morning or afternoon session)’ as between-subjects factors. Subsequently, data for the performance of the mice on their first day of training in Stages 2 (VITI schedule, *go* signals only) 3 and 4, (VITI, *go/no*‑*go* ratio of 2:1 and 5:1, respectively) of training were analysed using single measure ANOVA with ‘Genotype’ and ‘Time-of-Day’ as the between subjects factors. The number of daily sessions needed for the two genotypes to complete their training (‘*total sessions*’) was compared using repeated-measures ANOVA with ‘Genotype’ and ‘Time of Day’ as between-subjects factors.

Data from NI-1 were analysed using single measure ANOVA with ‘Genotype’ and ‘Time of Day’ as the between subjects factors. Where there was no main effect of Time of Day, this factor was collapsed on all other factors. Further analyses were conducted within the session whereby performance was divided into bins of trials (50 trials/bin). These bins were analysed using repeated-measures ANOVA with ‘Trial-Bin’ as the within‑subjects factor and ‘Genotype’ and ‘Time-of-Day’ as the between-subjects factors.

ANCOVA was also carried out to ascertain whether any genotype difference in *perseveration* could account for a (genotype-dependent) prolongation of the *latency to collect the reward*: *i.e.* the number of *perseverative* responses by individual mice was used as a covariate in the statistical analysis of its *latency to collect the reward*.

The *post-hoc* LSD test was used for pair-wise comparisons of the experimental groups. In all cases, statistical significance was set at *P *< 0.05.

## 3 Results

**Fig. 1:**
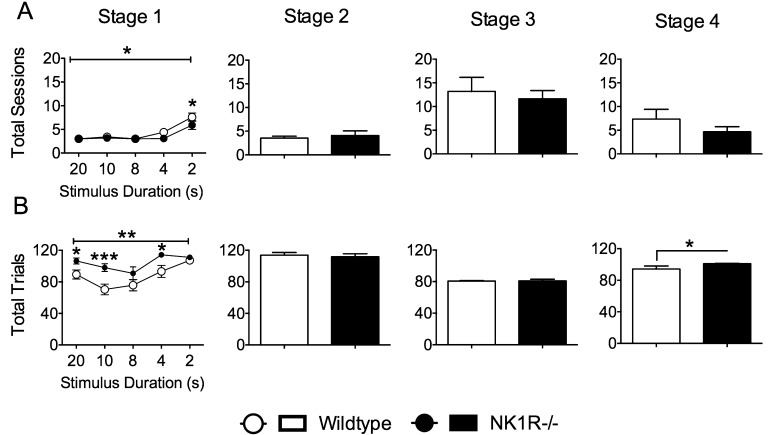
During training in the 5C-CPT, NK1R−/− mice needed fewer daily sessions to reach performance criteria and they completed more trials than their wildtypes. Circles/bars show mean ± s.e.m. For Stages 2–4, the bars show data for the first day of training, only. A: *total sessions* required to attain performance criterion at each stage of training; and B: *total trials* completed. Numbers on abscissae for Stage 1 specify the stimulus durations (SD: gradually reduced from 20 to 2 s as animals achieved progression criteria). A line above graphs/bar charts indicates an overall difference between NK1R−/− and wildtype mice. Statistical significance of differences between pairs of data (compared by genotype) indicated by: ^*^P < 0.05; ^**^P < 0.01; ^***^P < 0.001. *N *= 12 per group.

For most of the training procedure, there was no overall difference in the *total number of daily sessions* required by wildtype and NK1R−/− mice to complete each stage of training. However, NK1R−/− mice required slightly fewer sessions than wildtypes during Stage 1, especially during the final, most difficult, phase with the shortest SD [*F*_(1,22)_ = 4.99; *P *= 0.036] (Fig. 1A). Consistent with this slightly and transiently better competence in learning the task, the number of *total trials* completed by NK1R−/− mice during individual training sessions was higher than that by wildtypes for most of Stage 1 [*F*_(1,4)_ = 13.14; *P *= 0.001] and also Stage 4 [*F*_(1,22)_ = 5.43; *P *= 0.029] (Fig. 1B).

### 3.1 During training, NK1R−/−mice express more premature responses, but not false alarms, than wildtypes

**Fig. 2:**
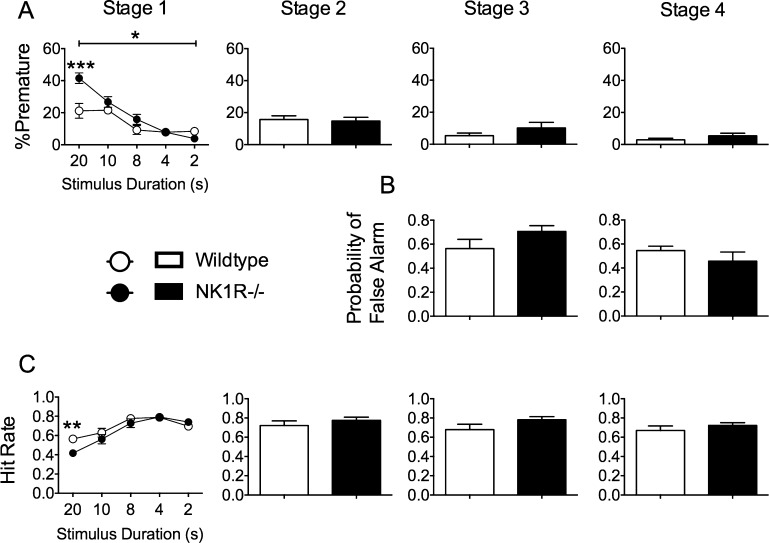
NK1R−/− mice carried out more *premature responses* and had a lower *hit rate* than wildtypes at the onset of training in Stage 1 of the 5C-CPT. Circles/bars show mean ± s.e.m. For Stages 2–4, the bars show data for the first day of training, only. A: % *premature responses*; B: *probability of false alarms*; and C: *hit rate*. Numbers on abscissae for Stage 1 specify the stimulus durations (gradually reduced from 20 to 2 s as animals achieved progression criteria). A line above graphs/bar charts indicates an overall difference between NK1R−/− and wildtype mice. Statistical significance of differences between pairs of data (compared by genotype) indicated by: ^*^P < 0.05; ^**^P < 0.01; ^***^P < 0.001. *N *= 12 per group.

During Stage 1 of training, NK1R−/− mice made more *premature responses* (an index of motor impulsivity), overall, than wildtype mice [*F*_(1,22)_ = 7.32; *P *= 0.013], especially during early exposure to the task (LSD: *P *< 0.001; Fig. 2A). On the first day of training in Stages 3 and 4, when the *no-go* signal was incorporated into the procedure, both genotypes carried out some *false alarms,* but there was no genotype difference for this measure of behavioural disinhibition (Fig. 2B). When first introduced to the training procedure [Stage 1], NK1R−/− mice carried out fewer cued plus uncued responses (‘*hit rate*’) than wildtype mice (genotype × stimulus duration): *F*_(4,80)_ = 3.09; *P *= 0.02; LSD *P *< 0.01) but there was no genotype difference in this measure at any subsequent stage of the task (Fig. 2C).

**Fig. 3:**
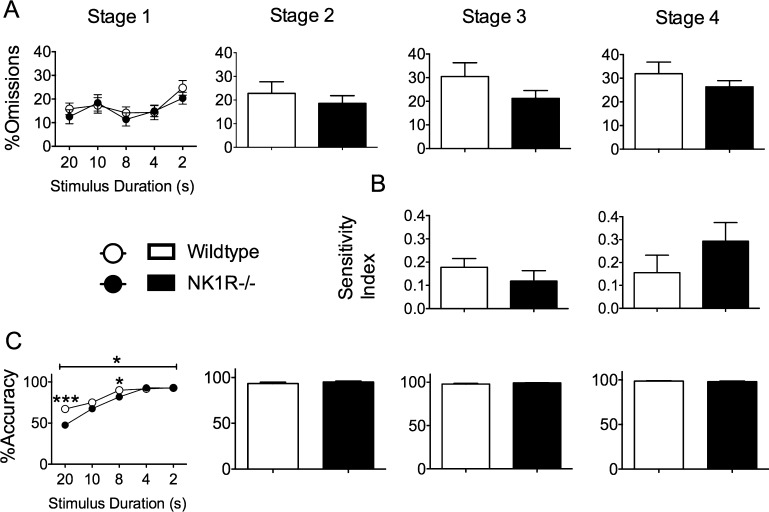
Attention in the 5C-CPT: NK1R−/− mice were less accurate than wildtypes at the onset of training in the 5C-CPT. Circles/bars show mean ± s.e.m. For Stages 2–4, the bars show data for the first day of training, only. A: *%omissions*; B: *sensitivity index;* and C: *%accuracy*. Numbers on abscissae for Stage 1 specify the stimulus durations (gradually reduced from 20 to 2 s as animals achieved progression criteria). A line above graphs/bar charts indicates an overall difference between NK1R−/− and wildtype mice. Statistical significance of differences between pairs of data (compared by genotype) indicated by: ^*^P < 0.05; ^**^P < 0.01; ^***^P < 0.001. *N *= 12 per group.

Neither *%omissions* (an index of inattentiveness) (Fig. 3A) nor the *sensitivity index* (an index of ‘vigilance’) (Fig. 3B) differed in the two genotypes on the first day of any stage of the training process. However, *%accuracy* (an index of selective attention) of NK1R−/− mice was slightly lower than that of the wildtypes during Stage 1, especially at the onset of training [*F*_(1,22)_ = 6.09; *P *= 0.022] (Fig. 3C).

**Fig. 4:**
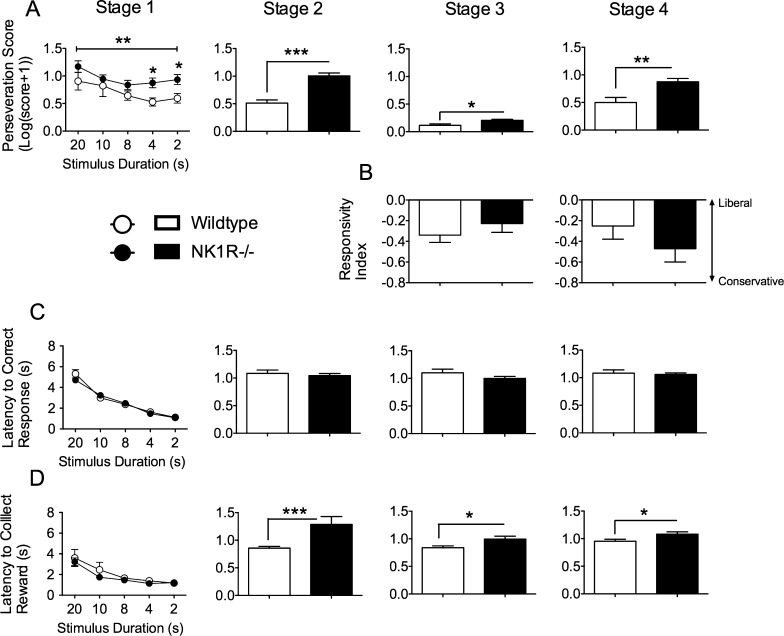
By comparison with wildtypes, NK1R−/− mice carried out more *perseverative responses* and had a longer *latency to collect their reward* during training and testing in the 5C-CPT. Circles/bars show mean ± s.e.m. A: *perseveration score*; B: *responsivity index*; C: *latency to correct response*; D: *latency to collect reward*. Numbers on abscissae for Stage 1 specify the stimulus durations (gradually reduced from 20 to 2 s as animals achieved progression criteria). A line above graphs/bar charts indicates an overall difference between NK1R−/− and wildtype mice. Statistical significance of differences between pairs of data (compared by genotype) indicated by: ^*^P < 0.05; ^**^P < 0.01; ^***^P < 0.001. *N* = 12 per group.

*Perseveration* was higher in NK1R−/− mice throughout training [Stage 1: *F*_(1,22)_ = 8.25; *P *= 0.009; Stage 2: *F*_(1,22)_ = 40.34; *P *< 0.001; Stage 3: *F*_(1,22)_ = 5.78; *P *= 0.025; Stage 4: *F*_(1,22)_ = 11.5; *P *= 0.003] (Fig. 4A). The *responsivity index* (an index of animals' tendency to respond to any light cue, which is a function of their motivation and innate response strategy) did not differ in the two genotypes (Fig. 4B). Consistent with this, there was no difference in *latency to correct response* at any stage of training (Fig. 4C). The *latency to collect the reward* was higher in NK1R−/− mice on the first day of Stages 2–4 (Fig. 4D) [Stage 2: *F*_(1,22)_ = 16.1; *P *< 0.001; Stage 3: *F*_(1,22)_ = 6.04; *P *= 0.022; Stage 4: *F*_(1,22)_ = 5.08; *P *= 0.034]. However, ANCOVA confirmed that this increase could be attributed to the higher *perseveration* score for NK1R−/− mice*,* which delayed their excursion to the magazine to collect their reward [*F*_(1,19)_ = 0.25; *P *= 0.63].

### 3.2 NK1R−/− mice do not carry out more false alarms, premature responses, or omissions during the extended test phase (NI-1) of the 5C-CPT

*Total trials* completed during the test session (NI-1) was again higher for NK1R−/− mice (213.4 ± 7.7) than wildtypes (178.6 ± 11.0).

**Fig. 5:**
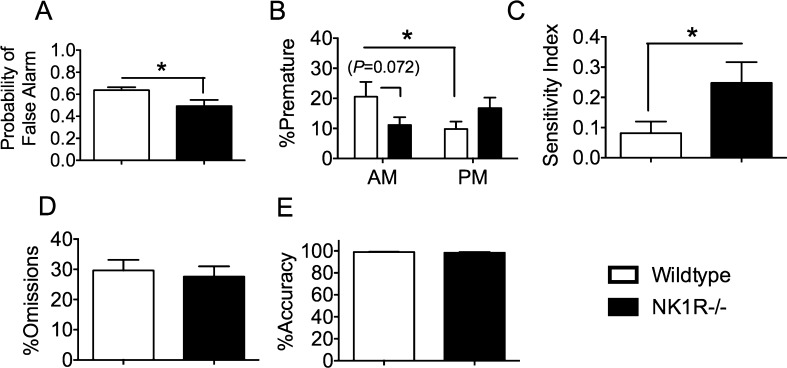
During the extended test session of the 5C-CPT (‘NI-1’), NK1R−/− mice expressed fewer *false alarms* and had a higher *sensitivity index* than wildtypes, but genotype differences in *premature responses* depended on time of day. Bars show mean ± s.e.m. Lines linking bars indicate statistically significant differences between groups. ^*^P < 0.05, ^**^P < 0.01, ^***^P < 0.001. *N* = 12 per group.

An apparently higher *hit rate* (*i.e.* total motor responses, whether or not promoted by a light cue) of NK1R−/− mice during NI-1 just missed the criterion for statistical significance (WT: 126 ± 10.8; NK1R−/−: 155 ± 9.4; *t*_(22)_ = 2.05; *P *= 0.052). There was a high incidence of *false alarms* for both genotypes but, contrary to our predictions, the *probability of a false alarm* during NI-1 was slightly lower in NK1R−/− mice than wildtypes [*F*_(1,22)_ = 5.31; *P *= 0.031] (Fig. 5A). There was also no overall genotype difference in the incidence of *premature responses.* However, as in our previous studies using the 5‑CSRTT, the incidence of this behaviour depended on time of day [Geno × Time-of-Day: *F*_(1,20)_ = 5.47; *P *= 0.03] (Fig. 5B): wildtypes produced fewer *premature responses* in the afternoon compared with the morning (LSD: *P *= 0.042) whereas there tended to be an increase in this behaviour by NK1R−/− mice. A genotype difference in *premature responses* in the morning just missed the criterion for significance (LSD: *P *= 0.072).

The *sensitivity index* was higher for NK1R−/− mice than wildtypes [*F*_(1,22)_ = 4.46; *P *= 0.046), suggesting that the former genotype was more vigilant, possibly even hypervigilant (Fig. 5C). Nevertheless, there was no genotype difference in either *%omissions* (*F*_(1,22)_ = 0.18; *P *= 0.679; Fig. 5D) or *%accuracy* [*F*_(1,22)_ = 0.33; *P *=0 .57] (Fig. 5E).

**Fig. 6:**
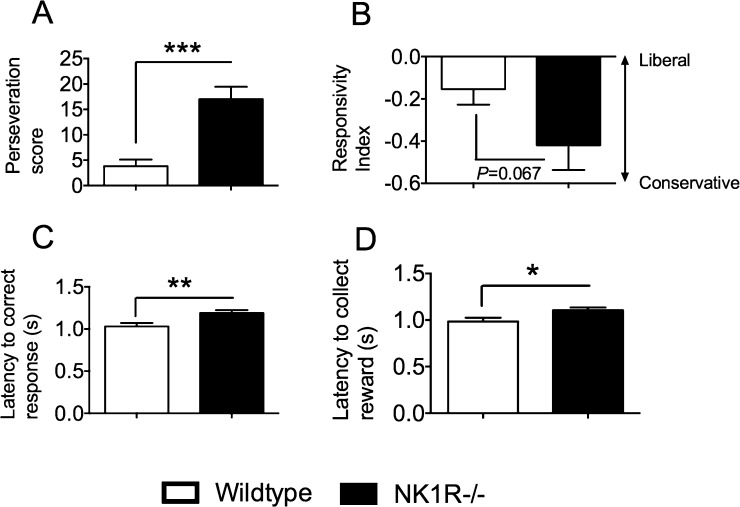
During the extended test session of the 5C-CPT (‘NI-1’), NK1R−/− mice had a higher *perseveration score* than wildtypes and a longer *latency to correct response* and *latency to collect their reward*. Bars show mean ± s.e.m. Lines linking bars indicate statistically significant differences between groups. ^*^P < 0.05, ^**^P < 0.01, ^***^P < 0.001. *N* = 12 per group.

Once again, *perseveration* was higher in NK1R−/− mice than wildtypes (*F*_(1,22)_ = 27.65; *P *< 0.001) (Fig. 6A). An apparent difference in the *responsivity index,* suggesting that NK1R−/− mice are more conservative about responding to the light signal, was not statistically significant [*F*_(1,22)_ = 3.72; *P *= 0.067] (Fig. 6B). Both the *latency to correct response* (*F*_(1,22)_ = 8.38; *P *= 0.008) (Fig. 6C) and *latency to reward* (*F*_(1,22)_ = 5.95; *P *= 0.023) (Fig. 6D) were increased in NK1R−/− compared to wildtype mice, albeit by only 0.1 s, approximately. However, as during training, there was no genotype difference in *latency to reward* when *perseveration* was treated as a covariate in the analysis (*F*_(1,19)_ = 0.25; *P *= 0.63).

### 3.3 Within test-session performance

**Fig. 7:**
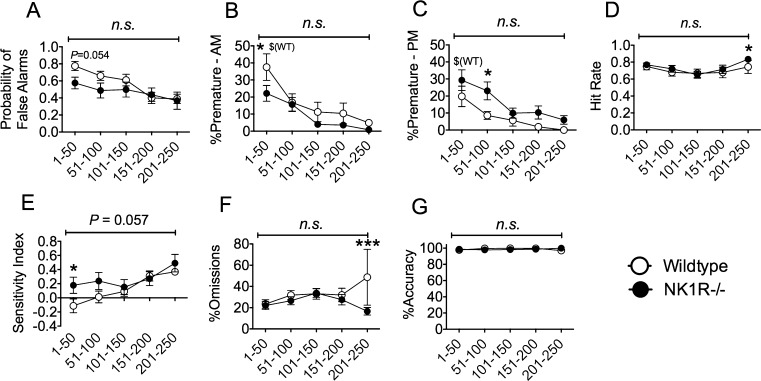
Changes in the performance of NK1R−/− mice and their wildtypes during the extended test session (NI-1) of the 5C-CPT. Circles show mean ± s.e.m. Numbers on the abscissae indicate bins of trials within NI-1. Although the performance of the two genotypes changed during NI-1, there was no overall genotype difference for any behavioural measure. With the exception of the *Probability of False Alarms* and *%Accuracy*, *post hoc* comparisons of pairs of data revealed differences between the two genotypes but in one trial-bin, only. ^*^P < 0.05; ^***^P < 0.001; (Genotype comparison) ^$^*P *< 0.05 (Time-of-Day comparison (AM *versus* PM)).

During the test challenge, in which the ITIs were increased, both the *probability of false alarms* [*F*_(4,71)_ = 4.26; *P *= 0.004] (Fig. 7A) and *%premature responses* [*F*_(4,64)_ = 30.72; *P *< 0.001] (Fig. 7B and C) decreased progressively through the NI-1 testing period. In the morning session, wildtypes expressed more *premature responses* during the first 50 trials of the test (*P *= 0.015) but this genotype difference was not seen in the afternoon because wildtype mice carried out fewer *premature responses* in these sessions (*P *= 0.006). As a consequence, NK1R−/− mice expressed more *premature responses* than wildtypes during trials 51–100 of the afternoon session (*P *= 0.023). Overall *hit rate* did not differ in the two genotypes, apart from during the last bin of trials when this measure was slightly higher in NK1R−/− mice (Fig. 7D).

The *sensitivity index* was higher in NK1R−/− mice than wildtypes during the first trial bin (1–50) but this difference dissipated subsequently (Fig. 7E). There was no difference in *%omissions,* overall, despite an apparent increase in wildtypes in the last time-bin (Fig. 7F). Finally, *%accuracy* was stable during the test and did not differ in the two genotypes (Fig. 7G).

## 4 Discussion

### 4.1 The ability to learn the 5C-CPT is not impaired in NK1R−/− mice

Mice lacking functional NK1R express behavioural abnormalities that are arguably analogous to those seen in ADHD. Here, we have compared the behaviour of NK1R−/− mice and their wildtypes in the 5C-CPT. This task presents both target and non-target stimuli, as do Continuous Performance Tests that are used to evaluate attention and impulsivity of ADHD patients.

The 5C-CPT has been used successfully in the past to study mice, including C57BL6 and DBA/2 strains [20]. Nevertheless, our first step was to ensure that NK1R−/− mice and their wildtypes could perform the test, which our findings confirmed. In fact, compared with wildtypes, NK1R−/− mice needed fewer sessions and completed more trials when training in Stages 1 and 4. This finding echoes evidence that the selective NK1R antagonist, SR140333, reverses stress-induced impairment of novel object/location recognition tests of rodents [32] and promoted inhibitory (step-through) avoidance of a footshock [33]. Furthermore, ADHD children show no evidence of learning deficits in a cued, instrumental‑learning task [32], [33], [34] provided that attentional demands are low [34], [35], [36], [37].

It is unlikely that a higher appetitive motivation explains why NK1R−/− mice completed more trials in this 5C-CPT. This is because there was no genotype difference in their *latency to respond.* Moreover, *latency to collect the reward* was longer in NK1R−/− mice than wildtypes, although this latter measure is confounded by a higher incidence of *perseveration* in NK1R−/− mice (see below), which delays their excursion to the reward magazine. Although NK1R−/− mice are smaller than wildtypes (see Section 2), there was no genotype difference in food consumption after correcting for differences in body size (unpublished results). Nevertheless, we acknowledge that other factors, not accessible to measurement in this 5C-CPT, could increase the motivation of NK1R−/− mice to perform the task.

### 4.2 NK1R−/− mice are not more impulsive than wildtypes during the test phase of the 5C-CPT

NK1R−/− mice carried out more *premature responses* than wildtypes only at the onset of Stage 1 of training in this 5C-CPT. It is interesting that this Stage resembles the 5-CSRTT in that it incorporates *go* but not *no-go*, signals. It is also notable that these inbred NK1R−/−mice typically carried out more *premature responses* in the 5-CSRTT when tested with a VITI, but not the LITI (see below) [11], [12], [15], [16]. One explanation for this disparity is that, in the 5-CSRTT, the signal latency (ITI) is constant during each stage of training and so the NI-1 test was the animals' first experience of an unpredictable ITI (VITI). By contrast, in this 5C-CPT, a VITI was used during both Stages 2–4 of training and the extended test session (NI-1). We have reported that a genotype difference in *premature responses* in the 5-CSRTT dissipates on repeated exposure to a VITI [40] and, in this study, the incidence of this behaviour declined even within the test session. Evidently, on repeated experience of the VITI, animals become more proficient at suppressing *premature responses*, as do ADHD patients [39], and this could abolish any genotype-dependent difference in this behaviour.

This evidence leads to the interesting possibility that a difference in *premature responses* by NK1R−/− mice and wildtypes in these tests depends on whether or not subjects expect the ITI to be unpredictable. Such a response contingency echoes a computational analysis [40], which inferred that a phasic response by noradrenergic neurones within the nucleus locus coeruleus signals a change in the predictability of a target, *go/no-go* stimulus (‘unexpected uncertainty’). Hence, it is also possible that the introduction of the non-target stimuli in Stage 3 of the 5C-CPT could have advanced this elevated noradrenergic response and changed the subsequent behaviour of the mice. There is also evidence that a deficit in functional NK1R impairs noradrenergic transmission in the mouse prefrontal cortex [6], [41], [42]. To the best of our knowledge, clinical studies have not compared the effects of such a change in signal predictability in CPTs on *premature responses* of ADHD patients and other subjects.

Two more potentially important differences in the protocols for this 5C-CPT and the 5-CSRTT could influence our findings. First, the NI-1 for the former test incorporated both an LITI and a VITI, whereas these were tested separately in the 5-CSRTT. Secondly, the duration of the VITIs used in the 5C-CPT (7–11 s) spanned a narrower time‑frame than those used in the 5‑CSRTT (2–15 s). Given that the likelihood of a *premature response* increases with longer ITIs, it is possible that only ITIs of duration ≥10–11 s distinguish a genotype difference in the number of *premature responses.*

Another finding was that *premature responses* in this 5C-CPT depended on whether the mice were tested in the morning or afternoon, as in the 5‑CSRTT when using the LITI [11], [15], [38]. Only when a VITI was used in the 5-CSRTT was there a higher incidence of *premature responses* by NK1R−/− mice, regardless of time of day [see above; [15], [16]]. Obviously, we cannot compare directly the findings from this study with those from the 5-CSRTT. However, collectively, they suggest that an influence of NK1R on *premature responses,* which depends on time of day, is evident only when the latency (LITI) of the target stimulus is consistently longer than anticipated on the basis of the training schedule.

It is well-known that, when conditioned stimuli are delivered with a fixed ITI, subjects use the time‑elapsed since the last stimulus to cue their response (‘interval-timing’). It follows that an error in interval-timing could contribute to an apparent increase in premature responses when testing with an LITI. Indeed, there are reports of errors in interval-timing by ADHD patients [43], [44], [45], [46], [47], [48] and that precision of interval-timing by humans follows a diurnal rhythm [49], [50]. This is interesting because this diurnal rhythm is disrupted in ADHD patients (*e.g.* [51]) as is the 24 h rhythm for the locomotor activity of NK1R−/− mice [12]. To the best of our knowledge, there are no reports that the frequency of premature responses in humans has a diurnal rhythm but this possibility should be taken into account when using a fixed ITI to compare ADHD patients with other groups of subjects.

By contrast, *false alarms* comprise an inappropriate response to a *no-go* signal, after it has been delivered and so will not be confounded by either errors in interval-timing or the schedule for delivery of the light cue (*i.e.* LITI *versus* VITI). However, NK1R−/− mice carried out fewer *false alarms* than wildtypes, during the extended test session. This finding does not necessarily undermine the validity of the NK1R−/− mouse in preclinical studies of ADHD. Despite reports that ADHD patients carry out more *false alarms* than controls (*e.g.* [52], [53], [54]), this is not a consistent finding (*e.g.* [55], [56], [57], [58], [59]) and the use of this behaviour to distinguish ADHD patients from other groups of subjects is controversial (see: [60]).

### 4.3 Inattentiveness (accuracy, omissions and sensitivity index)

In the 5‑CSRTT, *accuracy* is used as one index of attention [14], [61] and can be slightly impaired in NK1R−/− mice [[12], [15], but see: [16]]. Here, NK1R−/− mice were less accurate than wildtypes, but only at the start of training in Stage 1, suggesting that rehearsal of the task improves this aspect of attention. Whether or not *accuracy* is impaired in ADHD is unclear. One report suggests that ADHD patients do not exhibit lower *accuracy* in the human 5‑CSRTT [62] but recent evidence suggests that lower *accuracy* in ADHD could reflect an endophenotype [63].

Another index of attention, *%omissions*, which is typically higher in NK1R−/− mice when tested in the 5-CSRTT, did not differ in the test session (NI-1) of this 5C-CPT (*c.f.,* [12], [15]). This is possibly because of the animals' previous experience of the VITI, during training, ([see: [38]]) and/or the shorter stimulus latencies in this 5C-CPT, which will diminish the likelihood of an omission error. A third aspect of attention, the *sensitivity index* (an index of ‘vigilance’), was higher in NK1R−/− mice but this can be attributed to the lower incidence of *false alarms*.

Evidently, different measures of ‘attention’ are affected by a lack of functional NK1R in different ways in both the 5-CSRTT and the 5C-CPT. These disparities could give important clues to the role of NK1R on each of these aspects of attention.

### 4.4 Perseveration

*Perseveration* by NK1R−/− mice was higher than wildtypes throughout the procedure, as in most of our 5-CSRTT studies [15], [38], [64], [65]; but see [11], [12]. It is unlikely that the increased *perseveration* of NK1R−/− mice is explained by a higher rate of non-specific motor responses because their *hit rate* did not differ from wildtypes. Also, there was no genotype difference in the *responsivity index*, during either training or the test session, as has been reported for ADHD patients [66], also, albeit not consistently (*e.g.* [47]).

Isolation-reared rats similarly express increased *perseveration* in the 5-CSRTT and this was interpreted as reflecting cognitive inflexibility [67]. Some ADHD patients also express this behaviour in the Wisconsin Card Sorting Test [68] and Continuous Performance Tests [66] (*e.g.* [66]). In ADHD, *perseveration* is thought to reflect repetitive (compulsive) ‘checking’ to compensate for their attention deficit [69], [70]. Testing NK1R−/− mice in cross-species cognitive flexibility-specific tasks (*e.g.* [71], [72]) would help to interpret this element of the NK1R−/− mouse behavioural phenotype.

## Summary

NK1R−/− mice did not exhibit excessive *false alarms*, compared with wildtypes (response disinhibition) but, as in the 5-CSRTT, they exhibited more *perseverative responses* throughout the 5C-CPT. These results suggest that a subgroup of ADHD patients with polymorphism of the *TACR1* gene (the human equivalent of *Nk1r*) might similarly express more *perseveration*, but not *false alarms*, in Continuous Performance Tests. Furthermore, our findings point to certain procedural parameters, especially the latency and predictability of the light cue, that could be crucial for distinguishing the behavioural phenotypes of NK1R−/− mice and their wildtypes and, possibly, the characterisation of ADHD patients with polymorphism of the *TACR1* gene.

## Figures and Tables

**Table 1:** Stimulus parameters and progression criteria for training and testing in the 5‑CCPT.

Training		
**Stage**	**Schedule**	**Parameter**
**Habituation:**	All holes display a light signal	Continuous
Progression criteria: >70 reinforcers for 2 consecutive days		
**Stage 1:**	Fixed ITI *Go* trials, only	ITI: fixed at 5 s SD fixed: progressively reduced (s): 20, 10, 8, 4, 2 LH: 2 s longer than SD (min. 5 s)
Progression criteria (to progress from one SD to the next): • Mean correct latency (MCL) <0.5 SD for 3 consecutive days • At SD of 2 s, animals must achieve at least 10 correct responses for 3 consecutive days		
**Stage 2:**	Variable ITI (VITI) *Go* trials, only	ITI: 3,4,5,6 or 7 s; SD: 2 s; LH: 5 s
Progression Criteria: Accuracy >80: and %Omissions <40%		
**Stage 3:**	Variable ITI (VITI) 2:1 *Go*/*No-go* ratio: (80 *Go* trials + 40 *No-go* trials)	VITI: 3,4,5,6 or 7 s; SD: 2 s; LH: 5 s
Progression criterion: False Alarms (FA) <0.5		
**Stage 4:**	Variable ITI (VITI) 5:1 *Go*/*No-go* ratio: (100 *Go* trials + 20 *No-go* trials)	VITI: 3,4,5,6 or 7 s; SD: 2 s; LH: 5 s
Progression criterion: Sensitivity Index (SI) >0		
**Extended test session**		
	**Schedule**	**Parameters**
	250 trials (5:1 *Go*/*No-go* ratio)	VITI: 7,8,9,10,11 s; SD: 2 s; LH: 5 s

SD: Stimulus Duration (duration of the light stimulus). LH: Limited Hold (time allowed for a nose-poke response after the onset of the light stimulus). ITI: Intertrial Interval (time between the start of a trial and the onset of the light stimulus).

## References

[1] Kooij S.J., Bejerot S., Blackwell A., Caci H., Casas-Brugué M., Carpentier P.J., Edvinsson D., Fayyad J., Foeken K., Fitzgerald M., Gaillac V., Ginsberg Y., Henry C., Krause J., Lensing M.B., Manor I., Niederhofer H., Nunes-Filipe C., Ohlmeier M.D., Oswald P., Pallanti S., Pehlivanidis A., Ramos-Quiroga J.A., Rastam M., Ryffel-Rawak D., Stes S., Asherson P. (2010). European consensus statement on diagnosis and treatment of adult ADHD: the European Network Adult ADHD. BMC Psychiatry.

[2] Kieling R., Rohde L.A. (2012). ADHD in children and adults: diagnosis and prognosis. Curr. Top. Behav. Neurosci..

[3] American Psychiatric Association, Diagnostic and Statistical Manual of Psychiatric Disorders Fifth Edition++ (DSM-V), 2013.

[4] Arnold L.E., Hodgkins P., Kahle J., Madhoo M., Kewley G. (2015). Long-term outcomes of ADHD: academic achievement and performance. J. Atten. Disord..

[5] Harpin V.A. (2005). The effect of ADHD on the life of an individual, their family, and community from preschool to adult life. Arch. Dis. Child..

[6] Yan T.C., McQuillin A., Thapar A., Asherson P., Hunt S.P., Stanford S.C., Gurling H. (2010). NK1 (TACR1) receptor gene ‘knockout’ mouse phenotype predicts genetic association with ADHD. J. Psychopharmacol..

[7] Sharp S.I., McQuillin A., Marks M., Hunt S.P., Stanford S.C., Lydall G.J., Morgan M.Y., Asherson P., Curtis D., Gurling H.M. (2014). Genetic association of the tachykinin receptor 1 TACR1 gene in bipolar disorder, attention deficit hyperactivity disorder, and the alcohol dependence syndrome. Am. J. Med. Genet. B Neuropsychiatr. Genet..

[8] De Felipe C., Herrero J.F., O'Brien J.A., Palmer J.A., Doyle C.A., Smith A.J., Laird J.M., Belmonte C., Cervero F., Hunt S.P. (1998). Altered nociception, analgesia and aggression in mice lacking the receptor for substance P. Nature.

[9] Herpfer I., Hunt S.P., Stanford S.C. (2005). A comparison of neurokinin 1 receptor knock-out (NK1-/-) and wildtype mice: exploratory behaviour and extracellular noradrenaline concentration in the cerebral cortex of anaesthetised subjects. Neuropharmacology.

[10] Fisher A.S., Stewart R.J., Yan T., Hunt S.P., Stanford S.C. (2007). Disruption of noradrenergic transmission and the behavioural response to a novel environment in NK1R−/− mice. Eur. J. Neurosci..

[11] Porter A.J., Pillidge K., Grabowska E.M., Stanford S.C. (2015). The angiotensin converting enzyme inhibitor, captopril, prevents the hyperactivity and impulsivity of neurokinin-1 receptor gene ‘knockout' mice: sex differences and implications for the treatment of attention deficit hyperactivity disorder. Eur. Neuropsychopharmacol..

[12] Porter A.J., Pillidge K., Tsai Y.C., Dudley J.A., Hunt S.P., Peirson S.N., Brown L.A., Stanford S.C. (2015). A lack of functional NK1 receptors explains most, but not all, abnormal behaviours of NK1R−/− mice. Genes Brain Behav..

[13] Carli M., Robbins T.W., Evenden J.L., Everitt B.J. (1983). Effects of lesions to ascending noradrenergic neurones on performance of a 5-choice serial reaction task in rats; implications for theories of dorsal noradrenergic bundle function based on selective attention and arousal. Behav. Brain Res..

[14] Humby T., Wilkinson L., Dawson G. (2005). Assaying aspects of attention and impulse control in mice using the 5-choice serial reaction time task. Curr. Protoc. Neurosci..

[15] Yan T.C., Dudley J.A., Weir R.K., Grabowska E.M., Peña-Oliver Y., Ripley T.L., Hunt S.P., Stephens D.N., Stanford S.C. (2011). Performance deficits of NK1 receptor knockout mice in the 5-choice serial reaction-time task: effects of d-amphetamine, stress and time of day. PLoS One.

[16] Dudley J.A., Weir R.K., Yan T.C., Grabowska E.M., Grimmé A.J., Amini S., Stephens D.N., Hunt S.P., Stanford S.C. (2013). Antagonism of l-type Ca(v) channels with nifedipine differentially affects performance of wildtype and NK1R−/− mice in the 5-Choice Serial Reaction-Time Task. Neuropharmacology.

[17] Huang-Pollock C.L., Karalunas S.L., Tam H., Moore A.N. (2012). Evaluating vigilance deficits in ADHD: a meta-analysis of CPT performance. J. Abnorm. Psychol..

[18] Nichols S.L., Waschbusch D.A. (2004). A review of the validity of laboratory cognitive tasks used to assess symptoms of ADHD. Child Psychiatry Hum. Dev..

[19] Riccio C.A., Reynolds C.R. (2001). Continuous performance tests are sensitive to ADHD in adults but lack specificity. A review and critique for differential diagnosis. Ann. N.Y. Acad. Sci..

[20] Young J.W., Light G.A., Marston H.M., Sharp R., Geyer M.A. (2009). The 5-choice continuous performance test: evidence for a translational test of vigilance for mice. PLoS One.

[21] Young J.W., Powell S.B., Scott C.N., Zhou X., Geyer M.A. (2011). The effect of reduced dopamine D4 receptor expression in the 5-choice continuous performance task: separating response inhibition from premature responding. Behav. Brain Res..

[22] van Ekhuizen J., Acheson D., Risbrough V., Drummond S., Geyer M.A., Young J.W. (2014). Sleep deprivation impairs performance in the 5-choice continuous performance test: similarities between humans and mice. Behav. Brain Res..

[23] Barnes S.A., Young J.W., Neill J.C. (2012). Rats tested after a washout period from sub-chronic PCP administration exhibited impaired performance in the 5-Choice Continuous Performance Test (5C-CPT) when the attentional load was increased. Neuropharmacology.

[24] Turner K.M., Young J.W., McGrath J.J., Eyles D.W., Burne T.H. (2013). Cognitive performance and response inhibition in developmentally vitamin D (DVD)-deficient rats. Behav. Brain Res..

[25] Young J.W., Geyer M.A., Rissling A.J., Sharp R.F., Eyler L.T., Asgaard G.L., Light G.A. (2013). Reverse translation of the rodent 5C-CPT reveals that the impaired attention of people with schizophrenia is similar to scopolamine-induced deficits in mice. Transl. Psychiatry.

[26] McKenna B.S., Young J.W., Dawes S.E., Asgaard J.L., Eyler L.T. (2013). Bridging the bench to bedside gap: validation of a reverse-translated rodent continuous performance test using functional magnetic resonance imaging. Psychiatry Res..

[27] Tomlinson A., Grayson A.B., Marsh S., Harte M.K., Barnes S.A., Marshall K.M., Neill J.C. (2014). Pay attention to impulsivity: modelling low attentive and high impulsive subtypes of adult ADHD in the 5-choice continuous performance task (5C-CPT) in female rats. Eur. Neuropsychopharmacol..

[28] Evenden J.L. (1999). Varieties of impulsivity. Psychopharmacology (Berl.).

[29] Winstanley C.A., Dalley J.W., Theobald D.E., Robbins T.W. (2004). Fractionating impulsivity: contrasting effects of central 5-HT depletion on different measures of impulsive behaviour. Neuropsychopharmacology.

[30] Eagle D.M., Lehmann O., Theobald D.E., Pena Y., Zakaria R., Ghosh R., Dalley J.W., Robbins T.W. (2009). Serotonin depletion impairs waiting but not stop-signal reaction time in rats: implications for theories of the role of 5-HT in behavioural inhibition. Neuropsychopharmacology.

[31] Clark R.A., Shoaib M., Hewitt K.N., Stanford S.C., Bate S.T. (2012). A comparison of InVivoStat with other statistical software packages for analysis of data generated from animal experiments. J. Psychopharmacol..

[32] Kart-Teke E., Dere E., Brandão M.L., Huston J.P., de Souza Silva M.A. (2007). Reinstatement of episodic-like memory in rats by neurokinin-1 receptor antagonism. Neurobiol. Learn. Mem..

[33] Kart E., Jocham G., Müller C.P., Schlömer C., Brandão M.L., Huston J.P., de Souza Silva M.A. (2004). Neurokinin-1 receptor antagonism by SR140333: enhanced in vivo ACh in the hippocampus and promnestic post-trial effects. Peptides.

[34] Cheung C.H., Fazier-Wood A.C., Asherson P., Rijsdijk F., Kuntsi J. (2014). Shared cognitive impairments and aetiology in ADHD symptoms and reading difficulties. PLoS One.

[35] Luman M., Goos V., Oosterlaan J. (2015). Instrumental learning in ADHD in a context of reward: intact learning curves and performance improvement with methylphenidate. J. Abnorm. Child Psychol..

[36] Gitten J.C., Winer J.L., Festa E.K., Heindel W.C. (2006). Conditional associative learning of spatial and object information in children with attention deficit/hyperactivity disorder. Child Neuropsychol..

[37] Groen Y., Wijers A.A., Mulder L.J., Waggeveld B., Minderaa R.B., Althaus M. (2008). Error and feedback processing in children with ADHD and children with Autistic Spectrum Disorder: an EEG event-related potential study. Clin. Neurophysiol..

[38] Weir R.K., Dudley J.A., Yan T.C., Grabowska E.M., Peña-Oliver Y., Ripley T.L., Stephens D.N., Stanford S.C., Hunt S.P. (2014). The influence of test experience and NK1 receptor antagonists on the performance of NK1R−/− and wild type mice in the 5-choice serial reaction-time task. J. Psychopharmacol..

[39] Wilson T.W., Heinrichs-Graham E., White M.L., Knott N.L., Wetzel M.W. (2013). Estimating the passage of minutes: deviant oscillatory frontal activity in medicated and unmedicated ADHD. Neuropsychology.

[40] Yu A.J., Dayan P. (2005). Uncertainty, neuromodulation, and attention. Neuron.

[41] Yan T.C., Hunt S.P., Stanford S.C. (2009). Behavioural and neurochemical abnormalities in mice lacking functional tachykinin-1 (NK1) receptors: a model of attention deficit hyperactivity disorder. Neuropharmacology.

[42] Stanford S.C. (2014). Pschostimulants antidepressants and neurokinin-1 receptor antagonists (‘motor disinhibitors’) have overlapping, but distinct, effects on monoamine transmission: the involvement of l-type Ca2+ channels and implications for the treatment of ADHD. Neuropharmacology.

[43] Barkley R.A., Murphy K.R., Bush T. (2001). Time perception and reproduction in young adults with attention deficit hyperactivity disorder. Neuropsychology.

[44] McGee R., Brodeur D., Symons D., Andrade B., Fahie C. (2004). Time perception: does it distinguish ADHD and RD children in a clinical sample?. J. Abnorm. Child Psychol..

[45] Rubia K., Halari R., Christakou A., Taylor E. (2009). Impulsiveness as a timing disturbance: neurocognitive abnormalities in attention-deficit hyperactivity disorder during temporal processes and normalization with methylphenidate. Philos. Trans. R. Soc. Lond. B Biol. Sci..

[46] Allman M.J., Meck W.H. (2012). Pathophysiological distortions in time perception and timed performance. Brain.

[47] Kerns K.A., McInerney R.J., Wilde N.J. (2001). Time reproduction, working memory, and behavioural inhibition in children with ADHD. Child Neuropsychol..

[48] Barkley R.A., Koplowitz S., Anderson T., McMurray M.B. (1997). Sense of time in children with ADHD: effects of duration, distraction, and stimulant medication. J. Int. Neuropsychol. Soc..

[49] Bussi I.L., Levín G., Golombek D.A., Agostino P.V. (2014). Involvement of dopamine signaling in the circadian modulation of interval timing. Eur. J. Neurosci..

[50] Agostino P.V., do Nascimento M., Bussi I.L., Eguía M.C., Golombek D.A. (2011). Circadian modulation of interval timing in mice. Brain Res..

[51] Voinescu B.I., Szentagotai A., David D. (2012). Sleep disturbance, circadian preference and symptoms of adult attention deficit hyperactivity disorder (ADHD). J. Neural. Transm..

[52] Baijot S., Deconinck N., Slama H., Massat I., Colin C. (2013). Behavioural and neurophysiological study of attentional and inhibitory processes in ADHD-combined and control children. Acta Neurol. Belg..

[53] Durston S., Tottenham N.T., Thomas K.M., Davidson M.C., Eigsti I.M., Yang Y., Ulug A.M., Casey B.J. (2003). Differential patterns of striatal activation in young children with and without ADHD. Biol. Psychiatry.

[54] van der Stelt O., van der Molen M., Boudewijn G.W., Kok A. (2001). Neuroelectrical signs of selective attention to color in boys with attention-deficit hyperactivity disorder. Brain Res. Cogn. Brain Res..

[55] Johnstone S.J., Clarke A.R. (2009). Dysfunctional response preparation and inhibition during a visual *Go*/*No-go* task in children with two subtypes of attention-deficit hyperactivity disorder. Psychiatry Res..

[56] Smith J.L., Johnstone S.J., Barry R.J. (2004). Inhibitory processing during the *Go/No-Go* task: an ERP analysis of children with attention-deficit/hyperactivity disorder. Clin. Neurophysiol..

[57] Nagashima M., Monden Y., Dan Y.I., Dan H., Tsuzuki D., Mizutani T., Kyutoku Y., Gunji Y., Hirano D., Taniguchi T., Shimoizumi H., Momoi M.Y., Watanabe E., Yamagata T. (2014). Acute neuropharmacological effects of atomoxetine on inhibitory control in ADHD children: a fNIRS study. Neuroimage Clin..

[58] Börger N., van der Meere J. (2000). Motor control and state regulation in children with ADHD: a response study. J. Biol. Psychol..

[59] Kuntsi J., McLoughlin G., Asherson P. (2006). Attention deficit hyperactivity disorder. Neuromol. Med..

[60] Edwards M.C., Gardner E.S., Chelonis J.J., Schulz E.G., Flake R.J., Diaz P.F. (2007). Estimates of the validity and utility of the Conner’s continuous performance test in the assessment of inattentive and/or hyperactive-impulsive behaviours in children. J. Abnorm. Child Psychol..

[61] Hahn B., Shoaib M., Stolerman I.P. (2002). Nicotine-induced enhancement of attention in the five-choice serial reaction time task: the influence of task demands. Psychopharmacology (Berl.).

[62] Kuntsi J., Klein C. (2012). Intraindividual variability in ADHD and its implications for research of causal links. Curr. Top. Behav. Neurosci..

[63] Lin H.Y., Hwang-Gu S.L., Gau S.S. (2015). Intra-individual reaction time variability based on ex-Gaussian distribution as a potential endophenotype for attention-deficit/hyperactivity disorder. Acta Psychiat. Scand..

[64] Pillidge K., Porter A.J., Vasili T., Heal D.J., Stanford S.C. (2014). Atomoxetine reduces hyperactive/impulsive behaviours in neurokinin-1 receptor ‘knockout’ mice. Pharmacol. Biochem. Behav..

[65] Pillidge K., Porter A.J., Dudley J.A., Tsai Y.C., Heal D.J., Stanford S.C. (2014). The behavioural response of mice lacking NK_1_ receptors to guanfacine resembles its clinical profile in treatment of ADHD. Br. J. Pharmacol..

[66] Wu Y.H., Gau S.S., Lo Y.C., Tseng W.Y. (2014). White matter tract integrity of frontostriatal circuit in attention deficit hyperactivity disorder: association with attention performance and symptoms. Hum. Brain Mapp..

[67] Dalley J.W., Theobald D.E., Pereira E.A., Li P.M., Robbins T.W. (2002). Specific abnormalities in serotonin release in the prefrontal cortex of isolation-reared rats measured during behavioural performance of a task assessing visuospatial attention and impulsivity. Psychopharmacology (Berl.).

[68] Walshaw P.D., Alloy L.B., Sabb F.W. (2010). Executive function in pediatric bipolar disorder and attention-deficit hyperactivity disorder: in search of distinct phenotypic profiles. Neuropsychol. Rev..

[69] Mathews C.A., Jang K.L., Hami S., Stein M.B. (2004). The structure of obsessionality among young adults. Depress. Anxiety.

[70] Gurken K., Bilgic A., Turkoglu S., Kilic B.G., Aysev A., Uslu R. (2010). Depression, anxiety, and obsessive-compulsive symptoms and quality of life in children with attention-deficit hyperactivity disorder (ADHD) during three-month methylphenidate treatment. J. Psychopharmacol..

[71] Young J.W., Powell S.B., Risbrough V., Marston H.M., Geyer M.A. (2009). Using the MATRICS to guide development of a preclinical cognitive test battery for research in schizophrenia. Pharmacol. Ther..

[72] Young J.W., Geyer M.A. (2015). Developing treatments for cognitive deficits in schizophrenia: the challenge of translation. J. Psychopharmacol..

